# Insufficient iodine status in pregnant women as a consequence of dietary changes

**DOI:** 10.29219/fnr.v64.3653

**Published:** 2020-01-06

**Authors:** Solveig Adalsteinsdottir, Ellen Alma Tryggvadottir, Laufey Hrolfsdottir, Thorhallur I. Halldorsson, Bryndis Eva Birgisdottir, Ingibjorg Th. Hreidarsdottir, Hildur Hardardottir, Petra Arohonka, Iris Erlund, Ingibjorg Gunnarsdottir

**Affiliations:** 1 Unit for Nutrition Research, Landspitali University Hospital and Faculty of Food Science and Nutrition, University of Iceland, Reykjavik, Iceland; 2 Institution of Health Science Research, University of Akureyri and Akureyri Hospital, Akureyri, Iceland; 3 Centre for Fetal Programming, Department of Epidemiology Research, Statens Serum Institut, Copenhagen, Denmark; 4 Department of Obstetrics and Gynecology, Landspitali University Hospital, Reykjavík, Iceland; 5 Faculty of Medicine, University of Iceland Reykjavík, Iceland; 6 Forensic Toxicology Unit, National Institute for Health and Welfare, Helsinki, Finland

**Keywords:** Iodine, pregnancy, fish, dairy, supplements

## Abstract

**Background:**

Historically, Iceland has been an iodine-sufficient nation due to notably high fish and milk consumption. Recent data suggest that the intake of these important dietary sources of iodine has decreased considerably.

**Objective:**

To evaluate the iodine status of pregnant women in Iceland and to determine dietary factors associated with risk for deficiency.

**Methods:**

Subjects were women (*n* = 983; 73% of the eligible sample) attending their first ultrasound appointment in gestational weeks 11–14 in the period October 2017–March 2018. Spot urine samples were collected for assessment of urinary iodine concentration (UIC) and creatinine. The ratio of iodine to creatinine (I/Cr) was calculated. Median UIC was compared with the optimal range of 150–249 μg/L defined by the World Health Organization (WHO). Diet was assessed using a semiquantitative food frequency questionnaire (FFQ), which provided information on main dietary sources of iodine in the population studied (dairy and fish).

**Results:**

The median UIC (95% confidence interval (CI)) and I/Cr of the study population was 89 μg/L (42, 141) and 100 (94, 108) μg/g, respectively. UIC increased with higher frequency of dairy intake, ranging from median UIC of 55 (35, 79) μg/L for women consuming dairy products <1 time per week to 124 (98, 151) μg/L in the group consuming dairy >2 times per day (*P* for trend <0.001). A small group of women reporting complete avoidance of fish (*n* = 18) had UIC of 50 (21, 123) μg/L and significantly lower I/Cr compared with those who did not report avoidance of fish (58 (34, 134) μg/g vs. 100 (94, 108) μg/g, *P* = 0.041). Women taking supplements containing iodine (*n* = 34, 3.5%) had significantly higher UIC compared with those who did not take supplements (141 (77, 263) μg/L vs. 87 (82, 94), *P* = 0.037).

**Conclusion:**

For the first time, insufficient iodine status is being observed in an Icelandic population. There is an urgent need for a public health action aiming at improving iodine status of women of childbearing age in Iceland.

## Popular scientific summary

- Despite Iceland’s historically high iodine consumption, decreased fish and dairy consumption among women of reproductive age have been a concern.
- The median UIC of 89 μg/L in the present study indicates insufficient iodine status in the population of pregnant women in Iceland.
- Action needs to be taken on public health level to attend to the matter.

Iodine (I) is a trace mineral necessary for the production of thyroid hormone, thereby essential for a wide range of physiological processes such as proper growth, development, and metabolic function (1, 2). The requirement for iodine is significantly increased during pregnancy. Since iodine plays a critical role in brain development, its deficiency should be prevented during pregnancy (3). The effect of maternal mild to moderate deficiency has recently received more attention, especially with regard to offspring neurodevelopment (4, 5). A recent meta-analysis showed that insufficient iodine status (I:Cr < 150 μg/g), particularly in early pregnancy (before 14 weeks gestation), is related to lower verbal intelligence quotient (IQ) of the offspring (4, 5), and the tipping point for adverse effects on infant and toddler language skills has been suggested to be urinary iodine concentration (UIC) ~100 μg/L (6).

Historically, Iceland has been an iodine-sufficient nation due to notably high fish and milk consumption (7, 8). Due to this fact, the country does not have a strategy related to securing sufficient iodine status, such as fortification of salt. In 2009, the median UIC was 180 μg/L in a sample of pregnant women (*n* = 162) (9), which is in line with the recommended range of 150–249 μg/L defined by the World Health Organization (WHO) (10). The UIC of the women who did not comply with dietary recommendations of consuming fish at least two times per week and consecutively did not consume dairy products two times per day was lower than among those who reported following the dietary recommendations for both fish and dairy intake (160 μg/L compared to 220 μg/L, respectively) (9). However, the sample size was not large enough to assess the possible risk of iodine deficiency in groups consuming less fish and dairy. A more recent study on iodine intake among 183 pregnant women (at 20 weeks gestation) in Reykjavík from 2012 found that 25% of the participants had suboptimal iodine intake (11), and results from 2015 to 2016 suggest that the intake of important dietary sources of iodine has continued to decrease (12). Given the trends seen with decreased fish and dairy consumption of the pregnant population in Iceland, it is pivotal to monitor their iodine status to avoid adverse, nonreversible health effects on fetal development (4, 5). The aim of the present study was to evaluate the iodine status of the population of pregnant women in Iceland and to determine dietary factors associated with risk for deficiency.

## Methods

### Subjects

All women who visited the Prenatal Diagnostic Unit at Landspitali National University Hospital, Reykjavik, Iceland, in their 11–14th weeks of pregnancy between 2 October 2017 and 28 March 2018 were invited to participate in the study. During the study period (6 months), 1,684 women were scheduled for their first ultrasound screening at Landspitali, corresponding to approximately 77% of the pregnant population in Iceland. Out of these 1,684 women, 244 women (15%) were excluded from the study because they did not speak Icelandic and could therefore not fill out the questionnaire. Other exclusion factors included falling short or exceeding the 11–14 week pregnancy range, missing the scheduled appointment time, or miscarriage, which in total excluded an additional 90 women. This left a remaining 1,350 women eligible to participate in the study. Of these, 128 women declined because of personal time constraints, and 201 declined without further explanation. Therefore, 76% of eligible women (*n* = 1,021) agreed to participate in the study. Spot urine samples were provided by 73% of eligible women (*n* = 983).

The study was approved by the National Bioethics Committee (VSN-17-057-S1) and the Medical Directorate of Landspitali University Hospital (LSH 5-17). Written informed consent was obtained from the participants.

### Assessment of iodine status

Subjects were asked to complete a spot urine sample and instructed to collect the midstream of urine in a sterile screw-top container. Samples were stored in freezers at a temperature of −80°C until enough samples were collected for a group shipment. Concentration of iodine and creatinine in the urine samples was measured at the Biochemistry Laboratory at the National Institute for Health and Welfare (THL) in Helsinki (Finland). The laboratory (No.T077) has been accredited by Finnish Accreditation Service FINAS and it fulfills the requirement of the standard SFS-EN ISO/IEC 17025. The scope of accreditation covers the UIC method. The urine sampling and shipment was in accordance with EUthyroid project guidelines (13). UICs were assessed via inductively coupled plasma mass spectrometry (ICP-MS), using an Agilent 7800 ICP-MS system (Agilent Technologies Inc., Santa Clara, CA, USA). In brief, 100 μL of urine sample was extracted using ammonium hydroxide solution. Tellurium was used as an internal standard. On the ICP-MS, m/z = 127 was scanned for iodine determination. The National Institute of Standards and Technology (NIST) reference standard materials (SRM2670a and SRM3668 Level 1 and Level 2) were used to ensure the accuracy of the method. Coefficient of variation (CV) of control samples was 1.6–4.3%. The laboratory at THL participates regularly in the Ensuring the Quality of Urinary Iodine Procedures Program (EQUIP) organized by the Centers for Disease Control and Prevention (CDC). Urinary creatinine was determined by a fully automated direct enzymatic method using Abbott Architect ci8200 analyzer (Abbott Laboratories, Abbott Park, IL, USA). The CV of control samples was 1.0–1.1%. The laboratory participates regularly in the External Quality Assessment Scheme organized by Labquality (Helsinki, Finland).

According to WHO guidelines, a median UIC value between 150 and 249 μg/L is considered the optimal population range during pregnancy (14). The use of iodine to creatinine (I/Cr) ratio has been shown to adjust for variation in urine volume and dilution of spot samples (15). Iodine status is reported both as UIC (μg/L) and I/Cr (μg/g).

### Dietary intake and background

Women who agreed to participate in the study answered a questionnaire in an electronic format on dietary intake (food frequency questionnaire [FFQ]) and information regarding maternal age, education, smoking habits, parity, nausea in pregnancy, prepregnancy weight, and height. Information on weight and height was used to calculate prepregnancy body mass index (BMI) (kg/m^2^). BMI < 18.5 kg/m^2^ was defined as underweight, 18.5–24.9 kg/m^2^ as normal weight, 25–29.9 kg/m^2^ as overweight, and ≥30.0 kg/m^2^ as obese.

The FFQ assessed dietary habits through inquiring about the frequency of consumption of 30 different food items, beverages, and use of iodine-containing supplement. The first question in the FFQ was related to general avoidance or abstinence of specific food or food groups (including fish and dairy). When answering this question, women were instructed to provide information on their diet in general, and not to include avoidance only due to nausea or avoidance of raw fish during pregnancy. For other questions, the instructions were to record intake, reflecting dietary intake in the past 3 months (approximately from the beginning of pregnancy). Frequency of dairy intake was defined as 250 mL portions in the FFQ and intake of fish defined as fish consumed as a main meal. Women selected between 10 potential frequency responses ranging from ‘less than once a month’ to ‘more than 5 times a day’. The development of the FFQ has previously been described in detail and the FFQ published (12). The frequency responses for fish (lean and oily) and dairy intake (drinking milk and fermented dairy products, excluding cheese) were used to categorize women to compare UIC and I/Cr ratio across intake groups. According to the last national dietary survey, these two food groups provided 68% of the iodine in the Icelandic diet (16). Other individual food groups contributed to 8% (cheese) or less. Iodized salt is generally not available on the Icelandic market. Women were also asked how many portions of dairy they consumed the day before collection of the urine sample and also if they have had fish as a main meal. Responses for use of iodine containing supplements included ‘no’, ‘yes’, and ‘I don’t know’. Women who answered ‘yes’ or ‘I don’t know’ were asked to write the name of the supplement they were using, which was used to validate their answers.

### Statistical analysis

Data are presented as median and 95% confidence interval (CI) for median. Median UIC and I/Cr ratio between groups are compared using either Kruskal–Wallis H test for several independent samples or Mann–Whitney U test for two independent samples. Spearman’s correlation was used to assess trend in UIC and I/Cr across categories of dairy and fish intakes. The data were analyzed using IBM Statistical Package for Social Sciences (SPSS) for Windows, Version 24.0 (Armonk, NY, USA). The level of significance was accepted as *P* < 0.05.

## Results

The median UIC (95% CI) and I/Cr of the study population were 89 μg/L (42, 141) and 100 (94, 108) μg/g, respectively. Characteristics of subjects are detailed in Table 1, giving UIC and I/Cr in subgroups. Multiparous women had a higher UIC (*P* = 0.002) and I/Cr (*P* = 0.04) than nulliparous women, which could be explained by higher intake of dairy products by multiparous women compared with nulliparous women (data not shown). Maternal education, prepregnancy BMI, pregnancy-related nausea, and smoking during pregnancy were not related to iodine status. Median frequency of intake for dairy products (excluding cheese) was 1.1 times per day and for fish 1.3 times per week (lean fish 1 time per week and oily fish 0.3 times per week).

**Table 1:** Characteristics of the subjects and median UIC (μg/L) and I/Cr ratio (μg/g) in subgroups (*n* = 983^a^)

Characteristics	*n* (%)	UIC (95% CI)	I/Cr ratio (95% CI)
**Age**			
18–24 years	155 (15.8)	89 (74, 105)	84 (75, 98)
25–29 years	357 (36.3)	82 (75, 94)	97 (85, 109)
30–34 years	295 (30.0)	98 (88, 110)	122 (109,130)
35–39 years	146 (14.9)	94 (82, 104)	97 (88, 129)
40–45 years	27 (2.7)	61 (43, 231)	97 (59, 146)
		*P* = 0.27	*P* = 0.002
**Education**			
Elementary or lower	113 (11.5)	86 (77, 105)	92 (77, 115)
High school or trade school	290 (29.5)	92 (79, 107)	98 (86, 108)
Bachelor’s degree	334 (34.0)	89 (82, 99)	104 (90, 116)
Masters or doctorate degree	245 (25.0)	87 (84, 96)	109 (93, 126)
		*P* = 0.86	*P* = 0.29
**Parity**			
Nulliparous	434 (44.2)	79 (71, 87)	93 (85, 102)
Multiparous	549 (55.8)	98 (91, 106)	109 (97, 119)
		*P* = 0.002	*P* = 0.04
**BMI**			
<18.5 kg/m^2^	19 (2.0)	78 (24, 121)	80 (51, 178)
18.5–24.9 kg/m^2^	525 (53.9)	86 (78, 97)	99 (89, 109)
25.0–29.9 kg/m^2^	249 (25.6)	92 (86, 107)	107 (94, 122)
>30 kg/m^2^	181 (18.6)	89 (83, 96)	101 (94, 109)
		*P* = 0.15	*P* = 0.32
**Nausea**			
No	108 (11.0)	86 (67, 100)	96 (83, 110)
Yes, but never vomit	297 (30.2)	85 (79, 96)	104 (92, 115)
Yes, vomit sometimes	400 (40.7)	94 (82, 103)	102 (89, 113)
Yes, vomit daily	178 (18.1)	91 (82, 112)	97 (84, 107)
		*P* = 0.36	*P* = 0.96
**Smoking during pregnancy**			
No	938 (95.5)	89 (84, 97)	101 (95, 109)
Yes	44 (4.5)	87 (44, 156)	81 (60, 129)
		*P* = 0.89	*P* = 0.21

Data are presented as median and 95% CI for median. Median UIC and I/Cr ratio are compared between groups using either Kruskal–Wallis H test for several independent samples or Mann–Whitney U for two independent samples.

a Data are partly missing for the following variables: Age *n* = 2; BMI *n* = 9; smoking *n* = 1; education *n* = 1.

### Iodine status according to intake of fish and dairy

Iodine status in relation to intake of fish and dairy is shown in Table 2. A clear trend toward higher UIC and I/Cr ratio was seen with higher intake of dairy products, ranging from 55 μg/L and 53 μg/g for women consuming dairy products <1 time per week to 124 μg/L and 132 μg/g in the group consuming dairy >2 times per day, respectively (*P* for trend <0.001). Women were also asked how many portions of dairy they consumed yesterday (the day before collection of the urine sample), and clear trend toward higher UIC and I/Cr was seen with higher number of portions (Supplementary Table 1). Less than 20% of women consumed at least two portions of dairy daily (excluding cheese), and about 35% had two or more portions fish per week (Table 2). Women who reported avoidance or no consumption of dairy products had a UIC of 65 μg/L which was significantly lower than the UIC of 91 μg/L seen in the group who did not avoid dairy products (*P* = 0.012). A trend toward higher I/Cr was seen with more frequent consumption of fish (Table 2). We repeated the analysis separating categories of consumption of lean and oily fish (data not shown), and found for both similar trends as for total fish intake (not significant (NS) for UIC and significant for I/Cr). The small group of women who reported avoidance of fish (*n* = 18) had a lower I/Cr compared to those who did not (58 μg/L vs. 100 μg/L, respectively, *P* = 0.04), and the difference was indicative for UIC (*P* = 0.07). It should be noted that intake of fish was significantly lower in the group avoiding dairy and vice versa (Supplementary Table 2).

**Table 2:** The median urinary iodine concentration (UIC) (μg/L) and iodine to creatinine (I/Cr) ratio (μg/g) in relation to intake of fish and dairy

Intake	*n* (%)^a^	UIC (95% CI)	I/Cr ratio (95% CI)
**Frequency of dairy intake**			
Never to <1 a week	71 (7.2)	55 (35, 79)	53 (44, 64)
1 to <3 times a week	86 (8.9)	76 (64, 91)	73 (61, 88)
3 to <7 times a week	268 (27.7)	90 (75, 98)	90 (77, 99)
1 to <2 times a day	352 (36.4)	91 (82, 99)	116 (105, 124)
2 times a day or more	189 (19.6)	124 (98, 151)	132 (120, 145)
		*P* < 0.001	*P* < 0.001
**Frequency of fish intake**			
<0.5 times a week	132 (13.8)	79 (79, 101)	83 (62, 102)
0.5 to <1 times a week	160 (16.3)	80 (80, 114)	89 (78, 110)
1 to <2 times a week	346 (35.3)	81 (81, 104)	110 (94, 113)
2 times a week or more	342 (34.9)	76 (76, 97)	100 (94, 108)
		*P* = 0.63	*P* < 0.001
**Avoid dairy^b^**			
No	943 (95.9)	91 (85, 97)	103 (95, 109)
Yes	40 (4.1)	65 (40, 89)	62 (45, 97)
		*P* = 0.012	*P* = 0.002
**Avoid fish^b^**			
No	965 (98.2)	90 (84, 95)	100 (94, 108)
Yes	18 (1.8)	50 (21, 123)	58 (34, 132)
		*P* = 0.07	*P* = 0.04

Data are presented as median and 95% CI for median. Frequency of dairy intake was defined as 250 mL portions in the food frequency questionnaire and intake of fish defined as fish as a main meal (average portion size around 150 g). Median UIC and I/Cr ratio are compared between groups using Mann–Whitney U for two independent samples. Spearman correlation was used to assess trend in UIC and I/Cr across categories of dairy and fish intakes.

a Missing data: Frequency of dairy intake *n* = 17; frequency of fish intake *n* = 3.

b That is neither due to morning sickness nor avoidance of raw fish in pregnancy.

Only 17 women claimed that they were taking supplements containing iodine. However, after assessing the validity of answers (assessment of iodine content of the brand name provided), five out of these 17 women were not taking supplements containing iodine. Two women who claimed that they were taking supplements containing iodine wrote iron in the open answer box where women were asked to provide brand names. Altogether, 124 women did not know if the supplement they were taking contained iodine. Going through the list of brand names provided, 22 more women were labeled as taking supplements containing iodine. All women using iodine containing supplements claimed that they used the supplements at least five times per week and daily dose of most of the supplements contained 150 μg. As shown in Table 3, women who were taking iodine-containing supplements had significantly higher UIC and I/Cr ratio compared with those who did not (141 μg/L and 155 μg/g vs. 87 μg/L and 96 μg/g). No difference was seen in fish or dairy intake between supplement users and nonusers (Supplementary Table 1).

**Table 3:** Median UIC (μg/L) and I/Cr ratio (μg/g) according to reported use of iodine containing supplements (*n* = 864^a^)

Iodine supplements	*n* (%)	UIC (95% CI)	I/Cr ratio (95% CI)
No	830 (84.4)	87 (82, 94)	96 (90, 104)
Yes	34 (3.5)	141 (77, 263)	155 (119, 220)
		*P* = 0.04	*P* < 0.001

Data are presented as median and 95% CI for median.

a The question regarding iodine-containing supplements was added to the study when recruitment had started. Answers are missing for 119 subjects.

## Discussion

The median UIC of 89 μg/L indicates insufficient iodine status in the population of pregnant women in Iceland, using the cutoff value of 150 μg/L (14, 17, 18). Although cutoff values for severe and mild-to-moderate iodine deficiency during pregnancy have yet to be defined by the WHO (14), median urine concentration between 50 and 100 μg/L has been defined as mild-to-moderate iodine deficiency in the nonpregnant population and median UIC of 50 μg/L as iodine deficiency (18). No active strategies, such as fortification of salt, are in place in Iceland, and a very low percentage of women reported using supplements containing iodine in the present study (3.5%). Action needs to be taken on public health level to attend to the matter.

Despite Iceland’s historically high iodine consumption, decreased fish and dairy consumption among women of reproductive age have been a concern (9, 11, 19). In 2009, the UIC of pregnant women in the capital region in Iceland (*n* = 162) was in accordance with WHO recommendations with a median value of 180 μg/L (9), compared to the median UIC of 89 μg/L in the present study. It is difficult to directly compare these two studies as the previous study collected urine samples during the second or third trimester of pregnancy, while samples were collected close to the first trimester in the present study. Studies have shown intertrimester fluctuations in iodine excretion, either toward decreased excretion or increased (20, 21). However, the decrease seen in UIC in this 10-year period is much larger than that could be explained by the difference in study design alone. In the present study, 35% followed the Icelandic food-based dietary guidelines of consuming fish as a main meal at least two times per week, compared with 54% in the study in 2009 (9). In the study from 2009, cheese was included when reporting compliance to the food-based dietary guidelines on dairy intake, with 60% of women consuming at least two portions per day compared with 48% in the present study (data not shown). In the present study, we decided to exclude cheese from the dairy category as our preliminary data analysis suggested that the reported frequency of cheese did not contribute to iodine status. Other changes that might have occurred from the time of the last study include decreased proportion of haddock as the main fish species (which was 83% in the previous study from 2009). According to information in the Icelandic nutrition composition database, 100 g of raw haddock (lean fish) contains 191 μg of iodine. However, cod (lean fish), which is growing in popularity in the Icelandic diet, only contains 45 μg of iodine per 100 g. Likewise, freshwater oily fish species have little to no iodine. For example, salmon only contains 4 μg of iodine per 100 g (22). Unfortunately, we do not have data on fish species consumed in the present study (other than information that 70% of the fish consumed was lean fish, data not shown). Furthermore, there are numerous factors that can influence the iodine content in cow’s milk, including supplementation of cow fodder, source of iodine supplementation in feed, presence of iodine antagonists, and milk-processing practices (23, 24).

Out of the 18 women reporting avoidance of fish products, six women reported that they were allergic to fish (data not shown), and out of the 40 women who reported avoidance or abstinence of dairy products, 32 women reported either having milk allergy or lactose intolerance (data not shown). The results show that it is of great importance to provide information about iodine nutrition to women of reproductive age with diagnosed food allergies or intolerances for iodine rich foods and to those who consume very low amounts of fish and dairy. Part of the women who reported avoidance of dairy and fish might have been following a vegan or plant-based diet at the time of the study. It has been shown that vegan and plant-based diets during pregnancy can be safely adhered to, but adverse pregnancy outcomes are possible without educated meal planning to avoid nutrient deficiencies (25). Numerous studies have shown that individuals following a vegan or vegetarian diet have lower UIC than those following carnivorous diets (26–28). Plant-based diets and milk alternative options have become increasingly popular (29, 30).

Women using iodine-containing supplements in the present study had UIC close to the optimal range defined by the WHO, and as most of the iodine-containing supplements recorded were prenatal supplements, it is likely that they started taking the supplements postconception. Current research findings regarding benefits and effectiveness of beginning iodine supplementation during pregnancy remain inconclusive (31, 32), and it has been suggested that supplementation should begin before conception to be effective (6, 33).

When responding to the question ‘are you taking an iodine-containing supplement?’, 124 women (14% of those who were invited to answer the question) responded ‘I don’t know’, and despite very low intake of fish and dairy in a large proportion of the population studied, only 3.5% reported taking supplements containing iodine. Interestingly, the median intake of fish and dairy was not different between those who were taking supplements and those who did not. Yet, when examining the filled in answers detailing the supposed iodine supplement being taken, two women responded iron (may have been confused between the difference between iron and iodine), and other three women who thought that they were using supplements containing iodine turned out to be using supplements that did not contain iodine (an Icelandic prenatal supplement which does not contain iodine). The results of the present study highlight a lack of nutrition literacy, which is reflected in the knowledge of iodine in the population studied. Low iodine knowledge has been linked as a major risk factor for iodine deficiency (34–36). A study conducted in the United Kingdom and Ireland of women of childbearing age (*n* = 520) found, for example, that only 32% of the participants correctly identified pregnancy as the most critical life stage for adequate iodine nutrition and 41% were unaware of adverse health effects associated with deficiency (37). In Norway, the percentage of young women with low iodine knowledge scores has decreased from 75 to 40% in a recent reevaluation of iodine knowledge, which is likely attributable to increase attention on iodine by the Norwegian Health authorities and mass media coverage (35, 38).

The main strengths of the present study are large sample size (*n* = 983) and high participation rate (75%). According to Statistics Iceland, a total of 2,188 infants were born in Iceland from April to September 2018 (approximately covering the expected delivery dates of women attending 11–14 weeks ultrasound during the study period). The study therefore includes 45% of the total population of pregnant women in Iceland. It might be considered to be a limitation that recruitment was limited to the capital of Reykjavik. According to Statistics Iceland, about 70% of women in Iceland live in the capital area. However, we cannot exclude that iodine status of women living outside the capital area is different from that reported in the present study. Another limitation is that the study was conducted during the winter months. It is possible that seasonal variations could have influenced our results, but as studies have found that the iodine concentration in cow’s milk is lower in summer than during winter time (39, 40), sampling throughout the whole year might have resulted in even worse iodine status than in the present study. It should be noted that although a stronger association was found between intake of dairy and iodine status in the present study than with fish intake, this does not necessarily mean that dairy is a better source of iodine than fish in the population studied. A spot urine sample mainly reflects recent intake of iodine. Therefore, it is more likely when using this method to capture iodine from dietary sources consumed on a daily basis (as for dairy) than food consumed less frequently.

## Conclusion

Insufficient iodine status was observed for the first time in an Icelandic population, most likely related to a decline in fish and dairy intake. The reasons for the decrease in fish and dairy intake in Iceland (11, 12, 16) remain unknown. However, it could be speculated that public discussion on suggested negative health effects of dairy consumption and increased awareness of environmental aspects might have contributed. There is an urgent need for public health action aimed toward improving iodine status of women of reproductive age in Iceland. This might include actions toward increased fish and dairy intake, recommendations for use of supplements in high-risk groups, or use of iodized salt. Independent of which action will be selected by the Icelandic authorities, regular and systematic monitoring of iodine status needs to be implemented in Iceland.

## Supplementary Material

Insufficient iodine status in pregnant women as a consequence of dietary changes
