# Supplementary material for: Insufficient iodine status in pregnant women as a consequence of dietary changes
Source: Food Nutr Res. 2020 Jan 6;64:10.29219/fnr.v64.3653. doi: 10.29219/fnr.v64.3653 (PMC6958617; doi:10.29219/fnr.v64.3653)
Supplement: Insufficient iodine status in pregnant women as a consequence of dietary changes [file FNR-64-3653-s001.docx]

# Supplemental table 1. Median UIC (µg/L) and I/Cr ratio (µg/g) according to reported intake of dairy portions yesterday (the day before collection of urine sample).

|  | n (%) | UIC (95%CI) | I/Cr ratio (95%CI) |
| --- | --- | --- | --- |
| **Portions of dairy intake yesterday** |  |  |  |
| None | 188 (19.1) | 74 (56, 85) | 69 (63, 85) |
| 1 | 457 (46.5) | 87 (81, 95) | 92 (85, 102) |
| 2 | 242 (24.6) | 95 (83, 109) | 128 (117, 140) |
| 3 | 66 (6.7) | 124 (85, 137) | 126 (112, 147) |
| 4 or more | 30 (3.1) | 178 (118, 217) | 148 (113, 191) |
|  |  | *p<0.001* | *p<0.001* |

Data is presented as median and 95% Confidence Interval for Median. Spearman correlation was used to assess p-for trend between categories.

Supplemental table 2. The median (25^th^ and 75^th^ percentile) frequency of fish and dairy intake

|  | n (%) | Fish intake frequency/ week | Dairy intake frequency/day |
| --- | --- | --- | --- |
| **Frequency of dairy intake** |  |  |  |
| Never to < 1 a week | 71 (7.2) | 1.0 (0.4, 2.0) | 0.1 (0.1, 0.1) |
| 1 to < 3 times a week | 86 (8.9) | 1.3 (0.6, 2.0) | 0.2 (0.2, 0.3) |
| 3 to <7 times a week | 268 (27.7) | 1.1 (0.6, 2.0) | 0.7 (0.5, 0.8) |
| 1 to < 2 times a day | 352 (36.4) | 1.3 (0.8, 2.0) | 1.3 (1.1, 1.5) |
| 2 times a day or more | 189 (19.6) | 1.5 (0.8, 2.0) | 2.6 (2.2, 3.4) |
|  |  | *p=0.002* | *p<0.001* |
| **Frequency of fish intake** |  |  |  |
| < 0.5 times a week | 132 (13.8) | 0.0 (0.0, 0.2) | 1.1 (0.6, 1.6) |
| 0.5 to < 1 times a week | 160 (16.3) | 0.4 (0.4, 0.6) | 1.1 (0.6, 1.6) |
| 1 to < 2 times a week | 346 (35.3) | 1.1 (1.1, 1.5) | 1.1 (0 6, 1.8) |
| 2 times a week or more | 342 (34.9) | 2.6 (2.0, 3.0) | 1.1 (0.5, 1.8) |
|  |  | *p<0.001* | *p=0.007* |
| **Avoid Dairy** |  |  |  |
| No | 943 (95.9) | 1.3 (0.8, 2.0) | 1.3 (0.6, 1.8) |
| Yes | 40 (4.1) | 0.6 (0.0, 1.4) | 0.1 (0.0, 0.5) |
|  |  | *p<0.001* | *p<0.001* |
| **Avoid Fish** |  |  |  |
| No | 965 (98.2) | 1.3 (0.8, 2.0) | 1.1 (0.5, 1.7) |
| Yes | 18 (1.8) | 0.0 (0.0, 0.0) | 0.3 (0.1, 1.0) |
|  |  | *p<0.001* | *p=0.003* |
| **Iodine supplements** |  |  |  |
| No | 830 (84.4) | 1.3 (0.8, 2.0) | 1.1 (0.5, 1.7) |
| Yes | 34 (3.5) | 1.5 (0.8, 2.0) | 1.0 (0 5, 1.5) |
|  |  | *p=0.62* | *p=0.75* |

Frequency of fish and dairy intake was compared between groups using Mann-Whitney U for two independent samples. Spearman correlation was used to assess p-for trend between categories of dairy and fish intakes and frequency of fish and dairy intake. Frequency of dairy intake was defined as 250 ml portions in the food frequency questionnaire and intake of fish defined as fish as a main meal (average portion size around 150 g).
